# External Ventricular Drains and Infection Risk: Duration as the Dominant Predictor—A Systematic Review and Meta-Analysis

**DOI:** 10.3390/brainsci16050528

**Published:** 2026-05-15

**Authors:** Thamer H. Alsharif, Badr E. Hafiz, Lamair Albakri, Abdularhman D. Alofi, Ziad Alzahrani, Yazid Maghrabi, Moajeb Alzahrani

**Affiliations:** 1 Department of Neurosurgery, King Abdulaziz Specialist Hospital, Taif 26521, Saudi Arabia; 2 Department of Neurosciences, King Faisal Specialist Hospital and Research Centre, Jeddah 21499, Saudi Arabia; 3 Department of Neurosurgery, Makkah Health Cluster, Makkah 21955, Saudi Arabia; 4 Department of Neurosurgery, King Abdulaziz Medical City, Jeddah 21423, Saudi Arabia

**Keywords:** external ventricular drain (EVD), ventriculostomy-related infection, catheter duration, cerebrospinal fluid leak

## Abstract

**Highlights:**

**What are the main findings?**

• Prolonged external ventricular drain (EVD) duration (>10 days) is strongly associated with increased risk of ventriculostomy-related infection.
• Early EVD removal (≤10 days) reduces infection risk by approximately 55% (pooled OR 0.45).

**What are the implications of the main findings?**

• Clinicians should prioritize minimizing EVD duration and consider early removal or alternative CSF diversion strategies.
• Strict infection-control practices and early management of CSF leaks are essential to reduce EVD-related infections.

**Abstract:**

Background/Objectives: External ventricular drains (EVDs) are widely used in the management of intracranial hemorrhage and hydrocephalus; however, they carry a significant risk of device-related central nervous system infections, including ventriculitis and meningitis, which are associated with increased morbidity, mortality, and prolonged intensive care stays. We conducted a systematic review and meta-analysis to evaluate whether prolonged EVD duration (>10 days) is associated with an increased risk of ventriculostomy-related infection compared with shorter duration (≤10 days), and to explore the association with cerebrospinal fluid (CSF) leak where data were available. Methods: A comprehensive literature search of PubMed, Google Scholar, Web of Science, and Cochrane CENTRAL was performed from database inception through September 2025, including English-language clinical trials, cohort studies, and case–control studies reporting infection outcomes related to EVD management factors. Two reviewers independently screened studies and extracted data. A random-effects meta-analysis was conducted to calculate pooled odds ratios (ORs) with 95% confidence intervals (CIs). Results: Sixteen studies comprising approximately 5500 patients met the inclusion criteria. Shorter EVD duration (≤10 days) was associated with a significantly lower risk of infection (pooled OR 0.45, 95% CI 0.30–0.68; *p* = 0.0002), corresponding to a 55% reduction in the odds of ventriculostomy-related infection. Prolonged EVD duration was consistently associated with increased infection risk across studies. Conclusions:These findings suggest that minimizing EVD duration may reduce infection risk and support early removal when clinically feasible. However, given the observational nature of the included studies, the results should be interpreted with caution. Further research is warranted to evaluate additional modifiable risk factors, including CSF leakage and sampling practices.

## 1. Introduction

External ventricular drainage (EVD) is widely used to manage acute hydrocephalus and reduce intracranial pressure [1,2,3]. Common indications include subarachnoid or intraventricular hemorrhage, severe traumatic brain injury, hydrocephalus, and tumor-related mass effect. However, EVD placement carries a notable risk of central nervous system (CNS) infections, collectively referred to as EVD-related infections (EVDRI) [4]. Reported infection rates range from 5% to 20%, and even lower rates are associated with significant consequences, including meningitis or ventriculitis, prolonged intensive care stays, increased healthcare costs, and higher morbidity and mortality [3,5,6,7].

EVDRI primarily arise from bacterial contamination of the catheter. Biofilm formation on catheter surfaces is a key pathogenic mechanism, whereby bacteria adhere to the EVD and form biofilms that resist both host defenses and antimicrobial therapy. Additional sources of contamination include breaches in sterile technique during insertion or handling, repeated CSF sampling, and CSF leakage from skull defects or surgical wounds. Once bacteria enter the CSF, they can proliferate rapidly and cause deep-seated CNS infections that are difficult to eradicate. According to the Centers for Disease Control and Prevention (CDC), EVDRI are classified as organ/space surgical site infections (SSIs), including meningitis, ventriculitis, brain abscess, subdural or epidural infections, and encephalitis [8].

Multiple studies have investigated predictors of EVDRI, but findings remain inconsistent. An integrative review published in 2002, including both pediatric and adult populations, identified several risk factors for CSF infection—particularly within the first 10 days of catheterization—such as subarachnoid or intraventricular hemorrhage, skull fracture with CSF leak, craniotomy, systemic infection, and catheter irrigation [9]. More recently, a systematic review published in 2024 reported that existing studies are highly heterogeneous and generally of low quality, with risk factors largely related to EVD maintenance and management practices [10]. Proposed predictors include catheter dwell time, CSF leak, frequency of CSF sampling, and placement setting (intensive care unit versus operating room). However, the evidence remains conflicting, with some studies demonstrating a strong association between prolonged duration and infection risk, while others report no significant relationship [7,8].

Given these inconsistencies, we conducted an updated systematic review and meta-analysis to clarify the impact of key procedural factors on EVDRI. Specifically, we aimed to evaluate the association between EVD duration and CSF leak with the risk of infection across all eligible studies through September 2025.

### Objectives

The primary objective of this study was to assess the effect of EVD duration on infection risk. CSF leak was analyzed as a secondary factor when sufficient data were available, while other variables, such as CSF sampling frequency, were evaluated qualitatively and reported narratively.

## 2. Materials and Methods

This systematic review and meta-analysis (SRMA) was conducted in accordance with the PRISMA 2020 guidelines, and the completed PRISMA 2020 checklist is provided as Supplementary Material [11]. This review was not prospectively registered (e.g., PROSPERO), which represents a methodological limitation. However, the study was conducted in accordance with PRISMA 2020 guidelines, and predefined eligibility criteria and data extraction protocols were strictly followed to minimize bias. The study question was structured using the PICO framework (Table 1).

### 2.1. Information Sources and Search Strategy

A comprehensive literature search was conducted from database inception through September 2025 across PubMed, Google Scholar, Web of Science, and Cochrane CENTRAL. Although the search covered all available records, only studies published from January 2015 onward were included to reflect contemporary neurosurgical practice. The search strategy was developed using a combination of Medical Subject Headings (MeSH) and free-text terms. Boolean operators (AND/OR) were applied to construct a sensitive and inclusive search query.

An example of the final PubMed search strategy is provided below:

(“external ventricular drain” [Title/Abstract] OR “external ventricular drainage” [Title/Abstract] OR “ventriculostomy” [Title/Abstract] OR “EVD” [Title/Abstract]) AND (“infection” [Title/Abstract] OR “ventriculitis” [Title/Abstract] OR “meningitis” [Title/Abstract] OR “ventriculostomy-related infection” [Title/Abstract] OR “catheter-related infection” [Title/Abstract]) AND (“duration” [Title/Abstract] OR “drain duration” [Title/Abstract] OR “indwelling time” [Title/Abstract] OR “catheter duration” [Title/Abstract])

Although several major databases were included, additional databases such as Embase and Scopus were not searched, which may have resulted in omission of some relevant studies.

### 2.2. Screening of Studies

Study selection was performed in two stages. In the first stage, two independent reviewers (T.H. and B.H.) screened titles and abstracts for relevance based on the predefined eligibility criteria (Table 2). Studies that did not meet the inclusion criteria were excluded at this stage. When eligibility could not be determined from the abstract alone, the full text was retrieved for further evaluation.

In the second stage, full-text articles were independently evaluated by two reviewers [12]. Any disagreements during screening were resolved by discussion between the reviewers, and a third reviewer adjudicated unresolved conflicts.

### 2.3. Data Extraction

Data extraction was performed independently by two reviewers using a standardized form. Extracted variables included study characteristics (author, year of publication, country, and design), patient demographics (sample size, age group, and underlying diagnosis), and details of EVD management such as catheter duration, CSF sampling frequency, presence of CSF leak, and definition of infection. Any disagreements in data extraction were resolved by discussion, with a third independent reviewer adjudicating unresolved issues.

### 2.4. Risk of Bias Assessment

The risk of bias of the studies was assessed with the Newcastle-Ottawa Scale (NOS) [13]. The two reviewers carried out the evaluation independently, resolving any disagreements or concealment through a third reviewer. Discrepancies in risk-of-bias ratings were resolved by discussion, with a third independent reviewer adjudicating if necessary. Three studies with severe risk of bias were excluded from the final analysis. The quality of the included studies is assessed in Table 3.

### 2.5. Strategy for Data Synthesis

For data synthesis, statistical heterogeneity between included studies will be assessed using the chi-square test (χ^2^) and quantified with the I^2^ statistic. RevMan softwareRevMan 5.4.1 (2020) was used for analysis. Effect sizes were calculated according to the data type reported in each study. For dichotomous outcomes, pooled estimates were expressed as odds ratios (ORs) or risk ratios (RRs) with corresponding 95% confidence intervals (CIs). For continuous outcomes, standardized mean differences (SMDs) or mean differences (MDs) with 95% CIs were computed. Heterogeneity among studies was evaluated using the I^2^ statistic and Cochran’s Q test. To assess potential publication bias, funnel plots were visually inspected for asymmetry, and Egger’s regression test was performed to statistically evaluate small-study effects. When publication bias was suspected, the trim-and-fill method was applied to estimate the adjusted effect size.

## 3. Results

### 3.1. Study Selection and Screening

**Figure 1:**
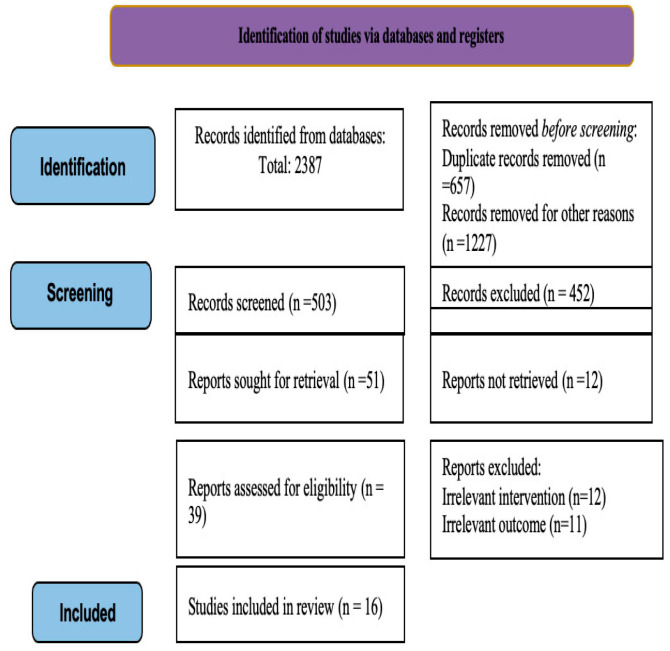
PRISMA Flow diagram of included studies.

A total of 2387 records were identified through database searching. After removal of duplicates and other reasons (1884], 503 records were screened by title and abstract. Of these, 39 full-text articles were assessed for eligibility. Ultimately, 16 studies were included in the systematic review and meta-analysis. The study selection process is illustrated in the PRISMA 2020 flow diagram (Figure 1) [29].

### 3.2. Study Characteristics

The included studies spanned from 2015 to 2024 and collectively enrolled approximately 5500 patients who underwent external ventricular drain (EVD) placement for diverse indications such as subarachnoid hemorrhage (SAH), intraventricular hemorrhage (IVH), traumatic brain injury (TBI), intracerebral hemorrhage (ICH), and tumor-related hydrocephalus. Most investigations were retrospective cohort or prospective observational designs; two incorporated randomized or controlled comparisons. Catheter types included standard, silver-impregnated, and antibiotic-impregnated EVDs. Definitions of infection varied (positive culture alone, clinical + laboratory criteria, or CDC definitions). Several studies also evaluated procedural factors such as CSF sampling frequency, CSF leak, and placement setting (ICU vs. OR). A summary of individual study characteristics is presented in Table 4.

### 3.3. Meta-Analysis

#### 3.3.1. EVD Duration

**Figure 2:**
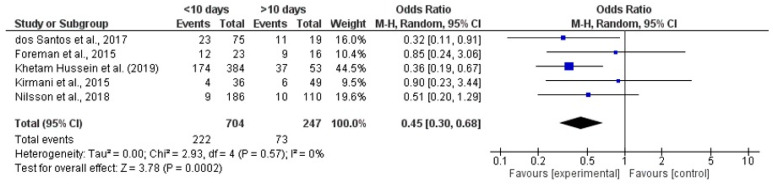
Forest Plot of Incidence of Infections in different EVD Duration [15,16,18,22,25].

A Forest Plot (Figure 2) of five studies was made using RevMan, evaluating the association between duration of external ventricular drainage (EVD) and risk of infection, revealed a significantly lower incidence of infection among patients with shorter EVD placement ((≤10 days) as compared to those with longer durations (>10 days). The pooled OR was 0.45 with a 95% CI of [0.30, 0.68] and *p* = 0.0002. This indicates that a shorter duration of EVD was associated with a 55% reduction in infection. No heterogeneity was detected among the studies (I = 0%).

#### 3.3.2. CSF Leak

**Figure 3:**
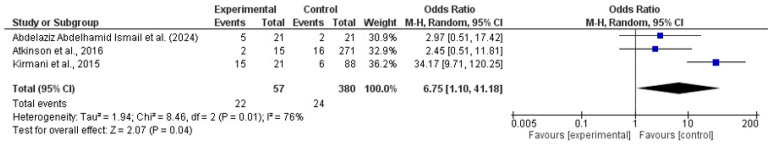
Forest Plot of CSF Leak [15,17,23].

Three studies (Figure 3) were used to plot a Forest Plot of CSF Leaks and incidence of infections. It showed that CSF Leaks were associated with a higher incidence of infections, and the results were significant. The pooled OR was 8.75 with a 95% CI of [1.10, 41.18] and *p* = 0.04. Although the result reached statistical significance, substantial heterogeneity (76%) was present, likely due to differences in leak definitions (e.g., wound leak vs. exit-site leak), catheter type, and infection-control practices.

#### 3.3.3. Publication Bias

**Figure 4:**
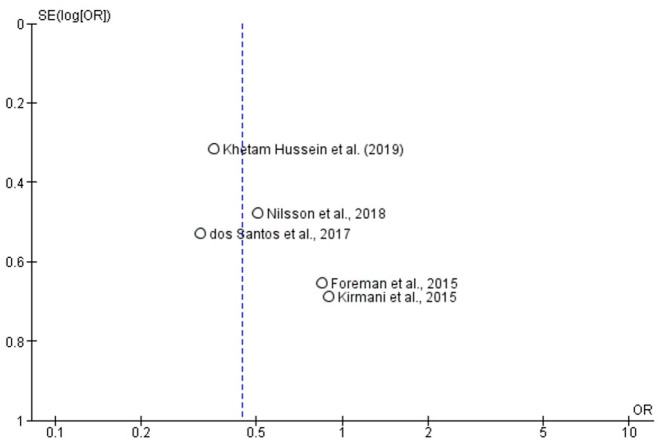
Funnel Plot of Publication Bias [15,16,18,22,25].

Visual inspection of the funnel plot (Figure 4) revealed a relatively symmetrical distribution of studies around the pooled effect estimate, suggesting a low likelihood of publication bias. The included studies were evenly dispersed on both sides of the mean effect line, with no substantial asymmetry indicating small-study effects. Furthermore, Egger’s regression test did not reveal statistically significant asymmetry (*p* > 0.05), supporting the absence of publication bias. Overall, these findings indicate that the results of this meta-analysis are unlikely to be influenced by selective reporting or small-study effects.

## 4. Discussion

This systematic review and meta-analysis identified prolonged EVD duration as the most consistent predictor of EVD-related infection (EVDRI). Patients with drains in place for more than 10 days had substantially higher infection rates compared with those with shorter durations. The pooled odds ratio (OR 0.45 for ≤10 days vs. >10 days) suggests a significant reduction in infection risk with earlier drain removal, with minimal statistical heterogeneity across studies. Clinically, these findings indicate that longer catheter dwell time increases the likelihood of infection. This is biologically plausible, as prolonged indwelling duration facilitates bacterial colonization, biofilm formation, and catheter-surface maturation, ultimately predisposing to ventriculitis [30,31].

Our findings are consistent with previous literature. The classic review by Lozier et al. (2002) reported that infection risk increases within the first 10 days of catheterization [9]. More recent cohort studies have similarly identified catheter duration as a key risk factor, with some reporting an approximate twofold increase in infection risk beyond 10 days. Recent reviews, including those by Sorinola et al. (2019) and De Andrade et al. (2024), have also highlighted catheter dwell time as a major modifiable risk factor [10,32].

The low heterogeneity observed in our analysis suggests consistency across studies; however, this finding should be interpreted with caution. The small number of included studies and similarities in study design or patient populations may have limited the detection of true heterogeneity. Therefore, the absence of statistical heterogeneity (I^2^ = 0%) does not necessarily indicate uniform effects across all clinical settings. From a clinical perspective, these results support minimizing EVD duration whenever feasible. Early weaning or transition to alternative CSF diversion strategies, such as ventriculoperitoneal shunting, should be considered to reduce infection risk.

### 4.1. Other Risk Factors

While duration emerged as the dominant risk factor, several other modifiable variables merit consideration.

#### 4.1.1. CSF Sampling Frequency

Frequent CSF sampling has been hypothesized to increase the risk of EVDRI by creating repeated opportunities for contamination. Some observational and case–control studies suggest a higher infection risk with frequent catheter manipulation, whereas others report minimal or no effect. In our review, a pooled analysis was not feasible due to inconsistent definitions and limited data. Notably, Thompson et al. (2018) found no significant increase in infection risk with daily sampling, suggesting that strict adherence to aseptic technique may mitigate this factor [28]. Nevertheless, minimizing unnecessary CSF access and maintaining strict sterile handling remain prudent clinical practices.

#### 4.1.2. CSF Leak

CSF leaks, including those from burr holes or skull defects, have long been recognized as a potential risk factor for infection [9]. In our analysis, CSF leak was associated with a higher incidence of infection; however, substantial heterogeneity was observed. This variability likely reflects differences in leak definitions, management strategies, and infection-control practices across studies. Clinically, these findings underscore the importance of early detection and repair of CSF leaks, meticulous wound closure, and regular site inspection to prevent ascending infection.

#### 4.1.3. Catheter Type

Several studies, including those by Atkinson et al. (2016), Foreman et al. (2015), and Nilsson et al. (2018) [16,17,22], suggest that antibiotic- or silver-impregnated catheters reduce infection risk by approximately 40–50% compared with standard silicone catheters. These devices likely inhibit early bacterial adhesion and biofilm formation. Although the number of studies was limited, future meta-analyses may be feasible, as multiple datasets report extractable infection outcomes by catheter type. However, potential heterogeneity related to coating material, infection definitions, and local antimicrobial practices should be considered.

#### 4.1.4. Placement Environment

EVD placement in the operating room under controlled sterile conditions has been associated with lower infection rates compared with bedside insertion in the ICU or emergency department, as demonstrated in studies by Foreman et al. (2015) and Altschul et al. (2020) [16,24]. This finding is biologically plausible, as operating room environments minimize contamination during insertion. Although limited data preclude robust quantitative synthesis, the available evidence supports preferential placement in controlled sterile settings when feasible.

#### 4.1.5. Infection-Control Bundles

Institutional infection-control bundles—including staff education, minimized catheter manipulation, daily assessment of necessity, and standardized insertion protocols—have been shown to significantly reduce infection rates. Studies by Hussein et al. (2019) and Walek et al. (2022) highlight the importance of system-level interventions in preventing EVDRI [3,25]. These findings suggest that adherence to structured protocols may be as important as individual procedural factors in reducing infection risk.

#### 4.1.6. Clinical Implications

The primary clinical implication of this study is that EVD duration is a key modifiable risk factor for infection. Clinicians should regularly reassess the necessity of EVD placement and remove the drain as soon as clinically feasible. When prolonged drainage is unavoidable, additional preventive strategies should be implemented, including the use of antibiotic-impregnated catheters and adherence to infection-control protocols. Transition to alternative CSF diversion methods, such as ventriculoperitoneal shunting, should be considered when appropriate.

Institutions should incorporate these findings into standardized EVD management protocols. For example, routine reassessment at 5–7 days may facilitate earlier decision-making regarding removal or alternative management. Limiting catheter manipulation to clinically necessary situations and ensuring strict aseptic technique are essential. Ongoing staff education and checklist-based approaches may further reduce infection rates.

#### 4.1.7. Study Limitations

This review has several limitations. Most included studies were observational and used heterogeneous definitions of EVD-related infection, ranging from positive CSF cultures to combined clinical, laboratory, and CDC-based criteria, which may affect comparability and pooled estimates. Although 16 studies were included, only a small subset contributed to the meta-analysis (five for EVD duration and three for CSF leak), limiting the precision of effect estimates and the ability to assess publication bias. This was largely due to variability in reporting and outcome definitions.

Additionally, device-related factors (e.g., catheter type or coating) and patient-level variables (e.g., age and comorbidities) were not analyzed in depth, as they were beyond the predefined scope of this study. Restricting inclusion to English-language studies may have introduced language bias. The inclusion of both adult and pediatric populations may also introduce clinical heterogeneity. Furthermore, the limited number of studies precluded subgroup or sensitivity analyses, including those based on infection definitions.

#### 4.1.8. Future Directions

Further research is needed to strengthen these findings. Large prospective multicenter studies or controlled trials, where feasible, should evaluate different EVD management strategies. Standardization of EVDRI definitions and consistent reporting of key variables—such as catheter duration, sampling practices, and CSF leak—are essential. Future studies should also explore interventions that mitigate duration-related risk, including advanced catheter technologies and targeted antimicrobial strategies. Evaluating patient-specific risk factors in combination with device-related variables may help identify high-risk populations and guide individualized management.

## 5. Conclusions

In this comprehensive review, we suggest that longer EVD placement is strongly associated with higher infection risk. Specifically, drainage beyond ~10 days was linked to significantly increased odds of ventriculitis: pooled analysis yielded an OR of 0.45 (95% CI 0.30–0.68) for EVD duration, implying about a 55% reduction in infection odds with shorter drainage. CSF leak appeared to raise infection risk in exploratory analysis. These findings reinforce that every additional week of EVD use increases infection risk, underscoring the importance of minimizing drain duration. Clinicians should prioritize early weaning or alternative CSF diversion when possible and maintain strict aseptic practices. By quantifying these risks, our review provides evidence to guide protocols aimed at reducing EVD-related infections. However, given the observational nature of the included studies and the absence of randomized controlled trials, these findings should be interpreted with caution, and higher-quality prospective studies are needed to confirm causality.

## Figures and Tables

**Table 1:** PICO Framework of SRMA.

P	Patients of any age who underwent external ventricular drain (EVD) placement for any indication
I	Prolonged duration of EVD
C	Shorter duration of EVD placement
O	EVD-related infection (ventriculostomy-associated infection, ventriculitis, or meningitis)

**Table 2:** Eligibility Criteria.

Characteristic	Inclusion Criteria	Exclusion Criteria
Language	English-language publications	Non-English publications
Time Period	January 2015–September 2025	NA
Study Design	Randomized controlled trials (RCTs), prospective or retrospective cohort studies, and case–control studies	Case reports, small case series (<10 patients), conference abstracts, editorials, commentaries, narrative reviews, or letters without original data
Population	Pediatric or adult patients who underwent external ventricular drain (EVD) placement for any clinical indication (e.g., hydrocephalus, subarachnoid hemorrhage, traumatic brain injury, intraventricular hemorrhage, meningitis)	Studies involving infections unrelated to EVD or following other neurosurgical procedures
Exposure/Intervention	Studies evaluating EVD duration (primary exposure) and other procedural factors (e.g., CSF leak, sampling frequency), which were analyzed qualitatively unless sufficient data were available for pooling.	Studies not assessing EVD duration, CSF sampling, or related procedural factors
Comparator	Shorter EVD duration (≤10 days) and/or less frequent CSF sampling	NA
Outcomes	Studies reporting incidence or risk of EVD-related infection (ventriculostomy-associated infection, ventriculitis, or meningitis)	Studies without defined or extractable infection outcomes
Context	Human studies evaluating EVD-related infection risk	Experimental or animal studies
Duplicates	Only the most complete or recent dataset included	Duplicate or overlapping datasets excluded

**Table 3:** NOS Quality Assessment.

Study	Selection (0–4)	Comparability (0–2)	Outcome (0–3)	Total Stars	Quality Level
Omar et al., 2015 [14]	★★★★	★	★★	7	High
Kirmani et al., 2015 [15]	★★★	★	★★	6	Moderate
Foreman et al., 2015 [16]	★★★	★	★★	6	Moderate
Atkinson et al., 2016 [17]	★★★★	★★	★★	8	High
dos Santos et al., 2017 [18]	★★★	★	★★	6	Moderate
Park et al., 2017 [19]	★★★	★	★★	6	Moderate
Woo et al., 2017 [20]	★★★★	★★	★★★	9	High
Kohli et al., 2018 [21]	★★★★	★	★★	7	High
Nilsson et al., 2018 [22]	★★★	★	★★	6	Moderate
Abdelaziz Abdelhamid Ismail et al., 2024 [23]	★★★	★	★★	6	Moderate
Konrad W. Walek et al., 2022 [3]	★★★★	★★	★★	8	High
David Altschul et al., 2020 [24]	★★★	★	★★	6	Moderate
Khetam Hussein et al., 2019 [25]	★★★★	★★	★★★	9	High
Feng Shang et al., 2018 [26]	★★★★	★	★★	7	High
Miki Katzir et al., 2019 [27]	★★★★	★	★★	7	High
Daniel Roan Thompson et al., 2018 [28]	★★★	★	★★	6	Moderate

The number of stars correlates with the overall rating as each star correlates with 1 as in the NOS.

**Table 4:** Characteristics of included studies.

Author and Year	Study Design	Location	Study Setting	Intervention	Cause of EVD	Population	Sample Size	Outcomes
Omar et al., 2015 [14]	Observational study	Peshawar, Pakistan	Neurosurgical Department MTI, Lady Reading Hospital	EVD placement (standard material used). CSF samples taken daily for 10 days (routine labs) and cultures taken on alternate day.	Intracranial hemorrhage (55.6% main indication), SAH, spontaneous/traumatic IVH, tumors associated with hydrocephalus, intraparenchymal bleed.	Consecutive patients who underwent EVD. Excluded: pre-existing intracranial infection, CSF leak, or revision of EVD.	18 consecutive patients	Frequency of EVD-related infections (defined by positive culture/Gram stain and supportive CSF findings).
Kirmani et al., 2015 [15]	Prospective study	Srinagar, Jammu and Kashmir, India	Neurosurgical service/Neurosurgical Intensive Care Unit (ICU)	Percutaneous ventriculostomy (EVD) insertion. Systemic antibiotics used prophylactically. CSF studies done at least once every 48 h. Catheter changes undertaken for blockage or routinely (commonly between 5th and 8th days).	SAHs (spontaneous/posttraumatic with/without hydrocephalus), hypertensive intracerebral bleeds with interventricular extensions. Majority: spontaneous SAH with IVH (55 cases), posttraumatic IVH (23).	Patients admitted to neurosurgical service requiring EVD. Excluded: prior shunt exteriorization, infection in first CSF sample, meningitis >7 days after EVD removal.	130 patients (193 ventriculostomies)	Overall infection rate (defined by positive culture OR biochemical criteria: CSF sugar < 15 mg/dL AND pleocytosis) and mortality. Risk factor analysis.
Foreman et al., 2015 [16]	Retrospective chart review	Birmingham, USA (Tertiary care center)	Intensive Care Unit (ICU) or Operating Room (OR)	EVD placement using antibiotic-impregnated catheters (minocycline and rifampin). Routine CSF sampling (Mon, Wed, Fri). Pre-placement antibiotics given in 100% OR patients vs. 10.8% ICU patients.	Head trauma (44.1% in ICU), SAH, IVH, Shunt malfunction/infection, Hydrocephalus unspecified, Tumor, ICH, CSF leak, Encephalocele.	Consecutive patients undergoing EVD placement (only first ventriculostomy evaluated).	138 patients (93 placed in ICU, 45 in OR)	Complications (hemorrhage, infection, non-functional drain) and accuracy of placement. Infection defined as positive CSF culture.
Atkinson et al., 2016 [17]	Retrospective analysis of prospectively collected data	Salford, UK (Tertiary referral centre)	Neurosurgery Department/University Teaching Hospital	Placement of Silver-impregnated EVD catheters (Silverline). Strict bundle of care approach used. Antibiotic prophylaxis (IV cefuroxime) administered. EVDs were not routinely changed or sampled. Catheter tunneled $\geq 6$ cm.	SAH, ICH, tumor, or trauma (TBI). Most EVDs placed in SAH patients.	Adult patients (>18 years) undergoing EVD placement. Excluded: existing or previous CSF infection.	263 individual patients (286 episodes)	Overall infection rate. Independent risk factors for CSF infection (defined by positive CSF culture OR clinical signs + positive blood culture).
dos Santos et al., 2017 [18]	Prospective, observational chart review study	Porto Alegre, RS, Brazil	Neurosurgery Department, Hospital Cristo Redentor (HCR)	EVD placement using standard Codman catheters. Antibiotic prophylaxis received by 76% of patients. CSF samples analyzed using CDC criteria.	Spontaneous intracranial hemorrhage: SAH (63%) or Hemorrhagic Stroke (HS) (37%).	Consecutive patients who required EVD for spontaneous intracranial hemorrhage.	94 consecutive patients	Infection rates (using CDC criteria for meningitis/ventriculitis) and mortality. Risk factor analysis.
Park et al., 2017 [19]	Prospective study	Daegu, Republic of Korea	Hospital setting/OR or ICU	EVD placement. IV cefazoline or vancomycin given before placement. CSF leak detection paper applied daily. CSF specimens collected 3 times a week (and as clinically indicated).	Acute hydrocephalus associated with SAH (n = 39) or IVH with intracerebral hemorrhage (n = 13). Excluded: head trauma, obstructive hydrocephalus, concomitant systemic infection.	Patients > 20 years who underwent EVD for acute hydrocephalus.	52 patients	Effect of CSF leak on infection and predisposing factors for CSF leak. Infection defined using strict criteria including CSF lab parameters and microbiological results.
Woo et al., 2017 [20]	Multicenter retrospective study	Seven neurosurgical centers in Hong Kong	Public healthcare service (Operating Theater setting)	Primary EVD placement. Catheters primarily plain silicon (99.8%). All procedures performed in OR under sterile conditions. Prophylactic systemic antibiotics administered to all patients.	Spontaneous intracerebral hemorrhage (32%), aneurysmal SAH (24%), traumatic brain injury (17%), Tumor (16%), Ischemic stroke (7%).	Patients who underwent primary EVD.	2575 patients	Incidence of VAI based on five definitions; identification of risk factors (using Gozal’s definition as primary endpoint).
Kohli et al., 2018 [21]	Retrospective, single-center study	Newark, New Jersey, USA	Emergency Department (ED), Intensive Care Unit (ICU), and Operating Room (OR)	EVD placement using non-antibacterial-impregnated catheters. Antibiotic prophylaxis given before placement and maintained throughout duration.	IPH (34.3%), SAH (13.5%), Neoplasm (17.2%), Trauma (7.98%), Primary hydrocephalus (14.7%).	Patients who underwent external ventriculostomy during hospitalization.	163 patients (190 EVDs)	Infection rate and complications. Infection defined as positive CSF culture.
Nilsson et al., 2018 [22]	Retrospective study	Lund, Sweden	Neurosurgical practice/Skåne University Hospital	Comparison between noncoated and Silver-coated EVD catheters (Silverline). EVDs placed in OR. Periprocedural antibiotics were not routinely used.	Spontaneous intracranial hemorrhage (68%), Trauma (18%), Brain tumor (9%).	Patients 18 years of age who received an EVD. Excluded: CNS infection at date of EVD insertion.	296 patients (186 noncoated, 110 silver-coated)	Rate of clinically diagnosed ventriculitis (defined as initiation of antibiotic treatment based on clinical suspicion).
Abdelaziz Abdelhamid Ismail et al. (2024) [23]	Prospective study comparing two randomized groups (Group A: VSGS; Group B: EVD)	Mansoura, Egypt (Mansoura University Hospitals)	Hospital/Neurosurgery Department	Comparison of EVD (Group B) vs. Ventriculosubgaleal shunt (VSGS) (Group A) management.	Post-Infective Hydrocephalus (PIH). (EVD Group B causes: 42.9% post meningitis, 42.9% post VPS infection, 14.2% post ETV infection).	Pediatric patients with PIH (excluded patients > 18 years old).	42 randomized cases (21 patients in Group B, EVD).	Comparison of complications, mortality rates, and cost/resource utilization (PICU admission, length of hospital stay (LOS)).
Konrad W. Walek et al. (2022) [3]	Retrospective review of prospectively collected data.	Providence, Rhode Island, USA	719-bed, tertiary-care, academic medical center (Neurocritical Care Unit utilized).	Implementation of a standardized infection control protocol prior to the study period, including minimal EVD manipulation, no routine CSF sampling, and regular cleansing.	Subarachnoid Hemorrhage (SAH) (30%), Intracerebral/Intraventricular Hemorrhage (ICH/IVH) (23%), Severe Traumatic Brain Injury (TBI) including EDH/SDH (23%), obstructive hydrocephalus, space-occupying lesion, AVM, or shunt failure.	Adult or pediatric patients.	409 patients, 479 EVDs.	Identify risk factors for EVD infections, overall infection rate, EVD duration, CSF leak, site dehiscence, and length of hospital stay (LOS).
David Altschul et al. (2020) [24]	Large retrospective study and analysis.	Bronx, New York, USA (single Comprehensive Stroke Center).	Emergency Room (ER) and Intensive Care Unit/Operating Room (ICU/OR).	Institutional protocol change (July 2015) shifting EVD placement location from the Emergency Room (ER) to the Intensive Care Unit/Operating Room (ICU/OR).	Stroke (Ischemic 1.7%, Hemorrhagic 54.83%—SAH, AVM, IPH, IVH, SDH) and Non-Stroke (43.47%—hydrocephalus, tumor bleeding, mass effect, VP shunts failure, TBI).	Patients of all ages.	710 patients.	Analyze incidence of EVDRI based on placement location (ER vs. ICU/OR) and assess the correlation between ERIs and subsequent need for VP shunt conversion.
Khetam Hussein et al. (2019) [25]	Prospective observational cohort study (included EVD, LD, and ICP drains).	Haifa, Israel (Rambam Health Care Campus).	Neurosurgical Intensive Care Unit (ICU).	Infection Control (IC) intervention bundle implemented March–August 2016 (focused on local guidelines for insertion/maintenance, isolation precautions, and staff education/adherence monitoring).	Devices inserted for: Hydrocephalus/increased ICP (28.4%), Trauma (38.2%), ICH (12.6%), SAH (10.3%), Infection (6.6%), and CSF leak (3.9%).	All consecutive adult patients undergoing insertion of EVD, ICP monitor, and LD.	232 patients with 437 drains (212 were EVDs).	Examine risk factors for meningitis/ventriculitis related to CSF drains; assess the effect of the IC intervention.
Feng Shang et al. (2018) [26]	Retrospective single center study.	Beijing, China (Xuanwu Hospital, Capital Medical University).	Neurosurgery Department, Intensive Care Unit (ICU).	Retrospective analysis of risk factors (duration, sampling frequency, antibiotics use).	SAH/ICH (75.9%), Intracranial tumors (10.3%), Others (5.2%).	Patients admitted to ICU of Neurosurgery Department.	58 patients.	Assess prevalence and potential risk factors of EVD-related infections, including duration of catheter drainage and CSF sampling frequency.
Miki Katzir et al. (2019) [27]	Retrospective cohort study comparing two periods/groups.	Haifa, Israel (Rambam (Maimonides) Health Care Campus).	Neurosurgical Intensive Care Unit (NSICU).	Protocol change (April 2013) switching from routine EVD replacement every 5 days (Group A) to replacement only when clinically indicated (Group B).	Head injury, Shunt malfunction, Aneurysmal SAH, IVH, ICH, Tumor with HCP, Stroke with HCP, Obstructive HCP.	Patients aged ≥18 years treated with an EVD for >5 days.	142 patients (Group A = 43; Group B = 99), 227 EVDs.	Evaluate the effect of the new protocol (clinically indicated replacement) on EVD-related infection rates; identify specific risk factors.
Daniel Roan Thompson et al. (2018) [28]	Retrospective, single-centre, age-matched, case–control study.	London, UK (King’s College Hospital).	Single-centre neurosurgical department.	Assessment of the association between recurrent EVD catheter sampling (exposure) and VAI (outcome), using a uniform management policy.	Cerebrovascular disease (e.g., SAH/ICH), Trauma, Vascular malformation, Tumour, CSF-related disorder.	Patients (excluding primary CNS infections, death within 5 days, and age < 1 year) who underwent EVD insertion.	83 cases (VAI) and 249 age-matched controls (Total of 332 patients analyzed).	Assess whether recurrent EVD sampling increased the risk of VAI; explore effect of sampling frequency, EVD duration, CSF leak, and concurrent systemic infections.

## Data Availability

No new data were created or analyzed in this study.

## Supplementary Materials

The following supporting information can be downloaded at https://www.mdpi.com/article/10.3390/brainsci16050528/s1, Table S1: 2020 Checklist.

## Abbreviations

The following abbreviations are used in this manuscript:

EVD	External Ventricular Drain
EVDRI	External Ventricular Drain-Related Infection
CSF	Cerebrospinal Fluid
SAH	Subarachnoid Hemorrhage
IVH	Intraventricular Hemorrhage
TBI	Traumatic Brain Injury
ICH	Intracerebral Hemorrhage
OR	Operating Room
ICU	Intensive Care Unit
CDC	Centers for Disease Control and Prevention
PRISMA	Preferred Reporting Items for Systematic Reviews and Meta-Analyses
NOS	Newcastle-Ottawa Scale
OR (statistical)	Odds Ratio
CI	Confidence Interval
